# Supplementary material for: Evidence for Non-Cancer-Specific T Cell Exhaustion in the Tcl1 Mouse Model for Chronic Lymphocytic Leukemia
Source: Int J Mol Sci. 2021 Jun 22;22(13):6648. doi: 10.3390/ijms22136648 (PMC8268419; doi:10.3390/ijms22136648)
Supplement: Supplementary file 1 [file ijms-22-06648-s001.zip › Supplement Figures.pdf]

**Supplemental figure legends**

**Fig S1. T cell populations, survival and tumor development of CLL-transplanted OT1/OT2, OT1 Rag/OT2 Rag and WT mice.** (A) Flow cytometry data of peripheral blood samples showing CD4/8 and Vb5$^{pos/neg}$ T cell distribution in non-transplanted OT1/OT2, OT1 Rag/OT2 Rag and WT mice. (B) Survival curves of CLL-transplanted OT1/OT2, OT1 Rag/OT2 Rag and WT mice and tumor development in OT1/OT2, OT1 Rag/OT2 Rag and WT mice depicted as percentage of lymphocytes.

**Fig S2. Depletion of T cells via pan B/CLL cell MACS isolation.** T cell (CD5$^{pos}$), B cell (CD19$^{pos}$) and CLL cell (CD5$^{pos}$ CD19$^{pos}$) abundance in spleen samples of CLL-transplanted (Tx) and non-transplanted (no Tx) WT mice before and post MACS.

**Fig S3. Endogenous T cell expansion in peripheral blood and spleen after CLL transplantation.** (A) Percentage of non-CLL specific (Vb5$^{pos}$) T cells in serial peripheral blood samples before and after tumor transfer in OT1/OT2, OT1 Rag/OT2 Rag and WT mice. (B) Endogenous T cell fractions (Vb5$^{neg}$) in spleen samples of non-transplanted vs. CLL-transplanted (Tx) OT1/OT2, OT1 Rag/OT2 Rag and WT mice.

**Fig S4. BCR analysis of CLL transplanted mice.** BCR clonality of CLL tumors 703, C755, and 658 used for transplantation experiments and of tumor-receiving WT, OT1/OT2, OT1 Rag/OT2 Rag mice. Color code corresponds to different BCR CDR3 sequences depicted in legends. BCR clones < 1% were bioinformatically excluded.

**Fig S5: PD1 expression based on flow cytometry, FACS and RNA-Seq analysis.** (A) Flow cytometry analysis of PD1 levels of transplanted WT, OT1 and OT2 mice. PD1$^{pos}$ fractions of WT CD4/CD8 (all Vb$^{pos}$), OT1 CD8 (Vb5$^{pos}$) and OT2 CD4 (Vb5$^{pos}$) T cells is depicted, respectively. Left panels represent PD1 isotype control, right panels show actual PD1 measurements. (B) Gating strategy for cell sorting of Vb5$^{pos/neg}$ PD1$^{pos/neg}$ populations is shown. (C) Normalized PD1 expression levels from RNA sequencing of sorted WT CD8/CD4 PD1$^{pos/neg}$ (n=3), OT1 CD8 PD1$^{pos/neg}$ (n=2 for Vb5$^{neg}$ populations, n=3 for Vb5$^{pos}$ populations) and OT2 CD4 PD1$^{pos/neg}$ (n=3) cell populations are shown. CPM: counts per million, FDR: false-positive detection rate

FIG S1

A

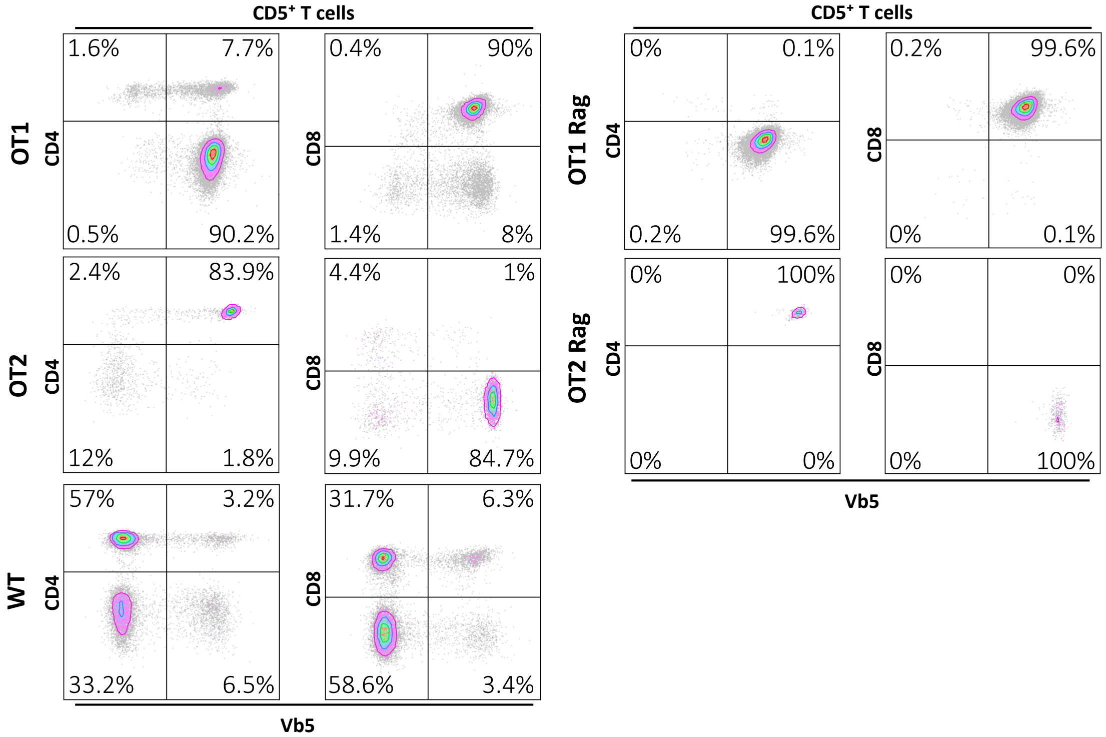


B

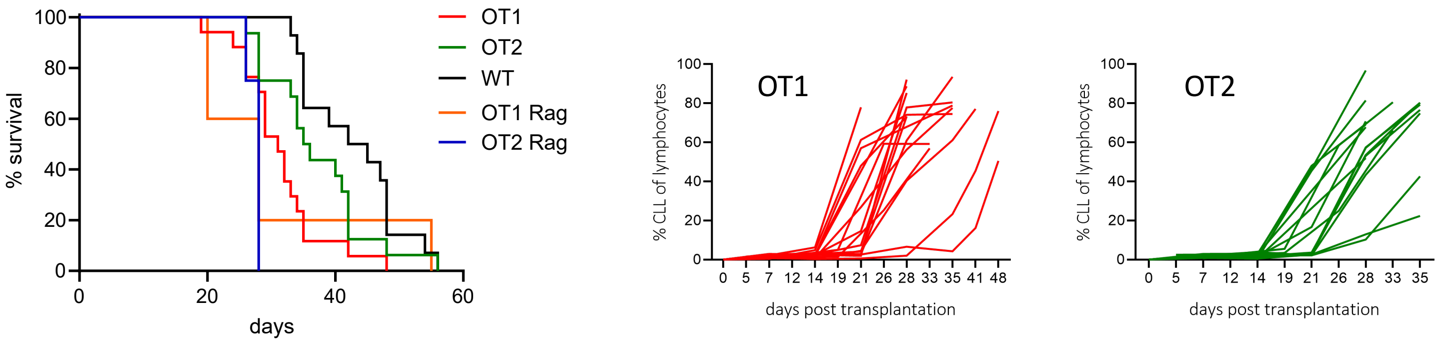


| Genotype comparison | p-value |
|---|---|
| OT1 vs OT2 | 0.0412 |
| OT1 vs WT | 0.0001 |
| OT2 vs WT | 0.0633 |
| OT1 vs OT1 Rag | 0.2138 |
| OT2 vs OT1 Rag | 0.0582 |
| OT1 vs OT2 Rag | 0.0865 |
| OT2 vs OT2 Rag | 0.0066 |
| WT vs OT1 Rag | 0.0124 |
| WT vs OT2 Rag | 0.0001 |
| OT1 Rag vs OT2 Rag | 0.7893 |

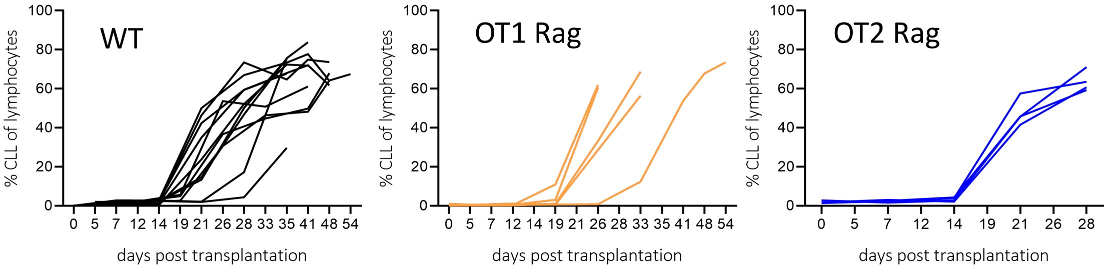

FIG S2

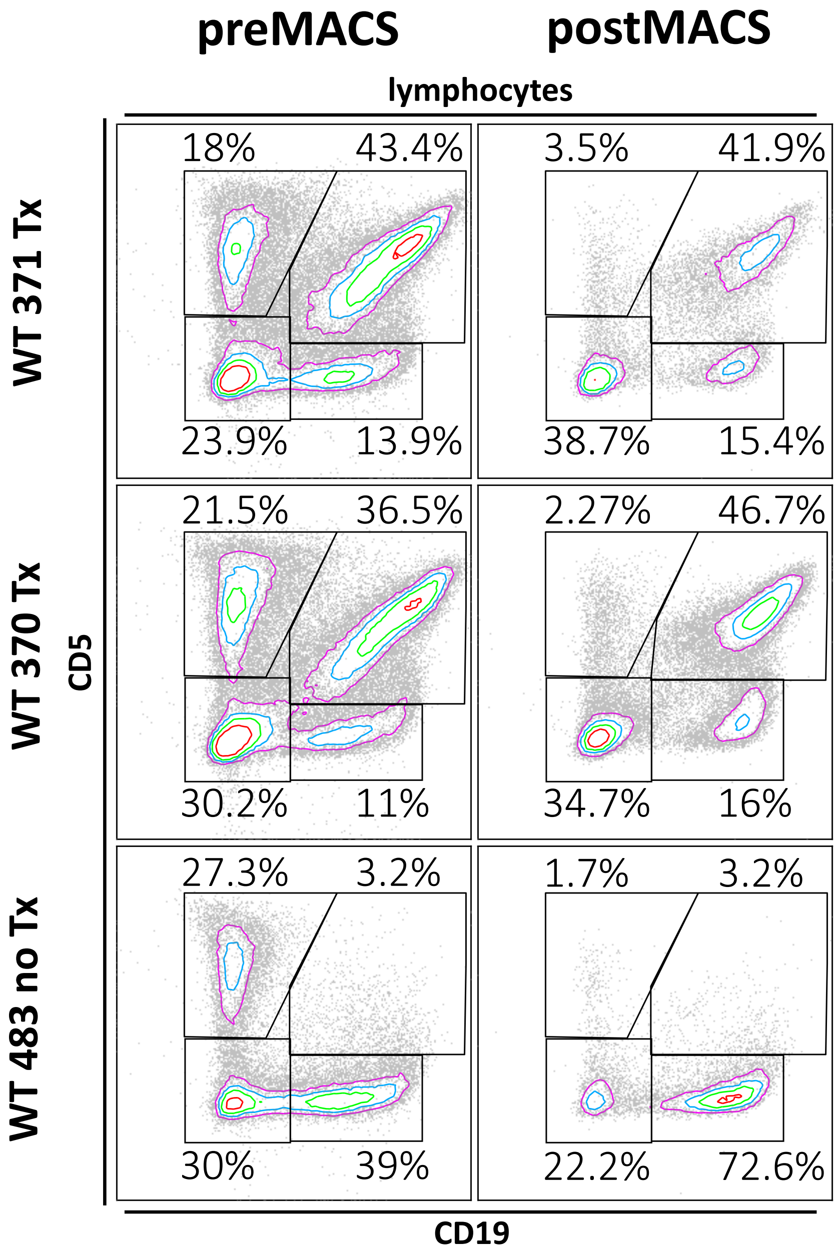
preMACS
postMACS
lymphocytes
WT 371 Tx
18%
43.4%
23.9%
13.9%
3.5%
41.9%
38.7%
15.4%
WT 370 Tx
21.5%
36.5%
30.2%
11%
2.27%
46.7%
34.7%
16%
WT 483 no Tx
27.3%
3.2%
30%
39%
1.7%
3.2%
22.2%
72.6%
CD5
CD19

FIG S3

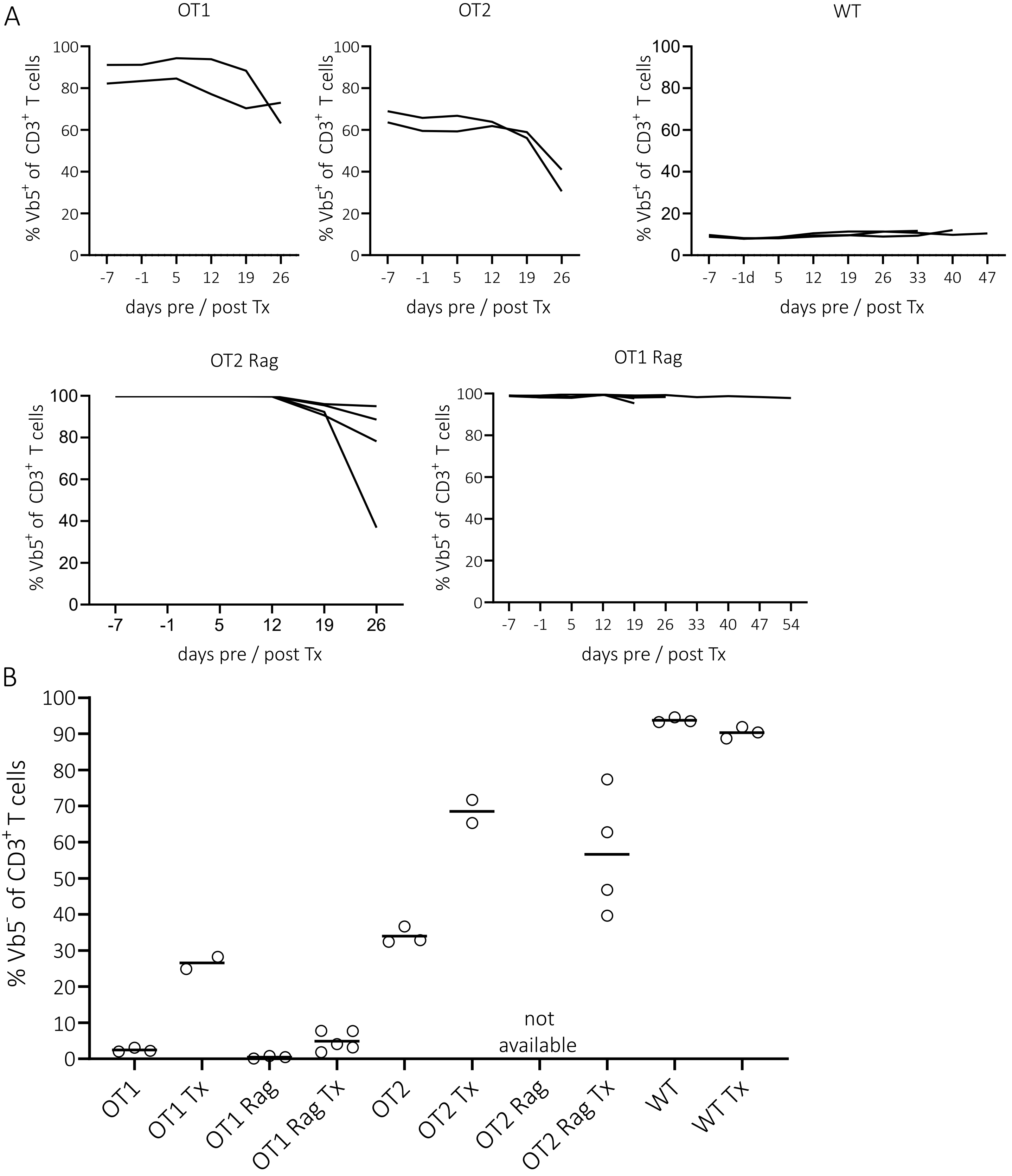

A
OT1
OT2
WT
% Vb5+ of CD3+ T cells
days pre / post Tx
OT2 Rag
OT1 Rag
B
% Vb5- of CD3+ T cells
not available
OT1
OT1 Tx
OT1 Rag
OT1 Rag Tx
OT2
OT2 Tx
OT2 Rag
OT2 Rag Tx
WT
WT Tx

FIG S4

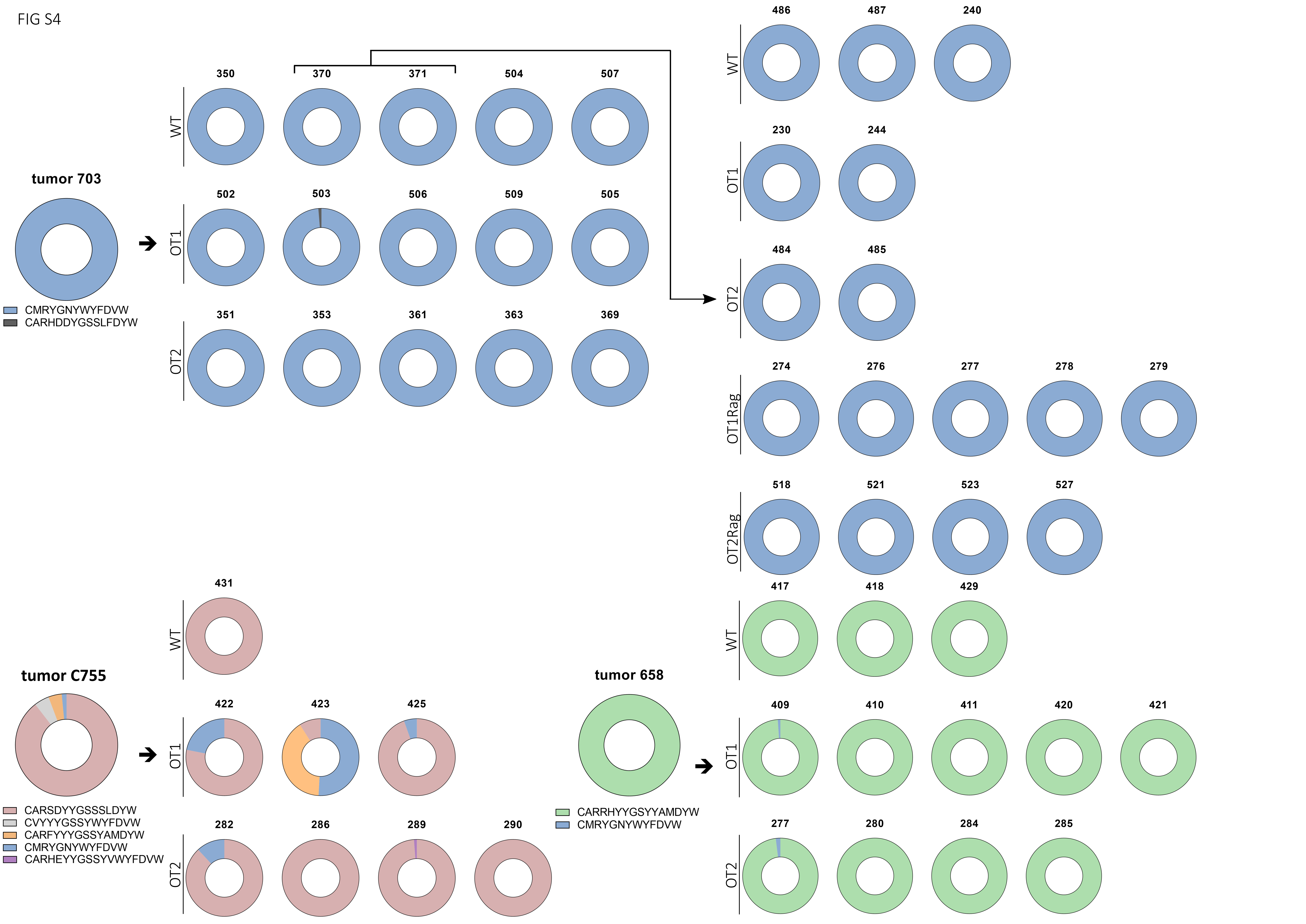

tumor 703
CMRYGNYWYFDVW
CARHDDYGSSLFDYW
WT
350
370
371
504
507
OT1
502
503
506
509
505
OT2
351
353
361
363
369
WT
486
487
240
OT1
230
244
OT2
484
485
OT1Rag
274
276
277
278
279
OT2Rag
518
521
523
527
tumor C755
CARSDYYGSSSLDYW
CVYYYGSSYWYFDVW
CARFYYYGSSYAMDYW
CMRYGNYWYFDVW
CARHEYYGSSYVWYFDVW
WT
431
OT1
422
423
425
OT2
282
286
289
290
tumor 658
CARRHYYGSYYAMDYW
CMRYGNYWYFDVW
WT
417
418
429
OT1
409
410
411
420
421
OT2
277
280
284
285

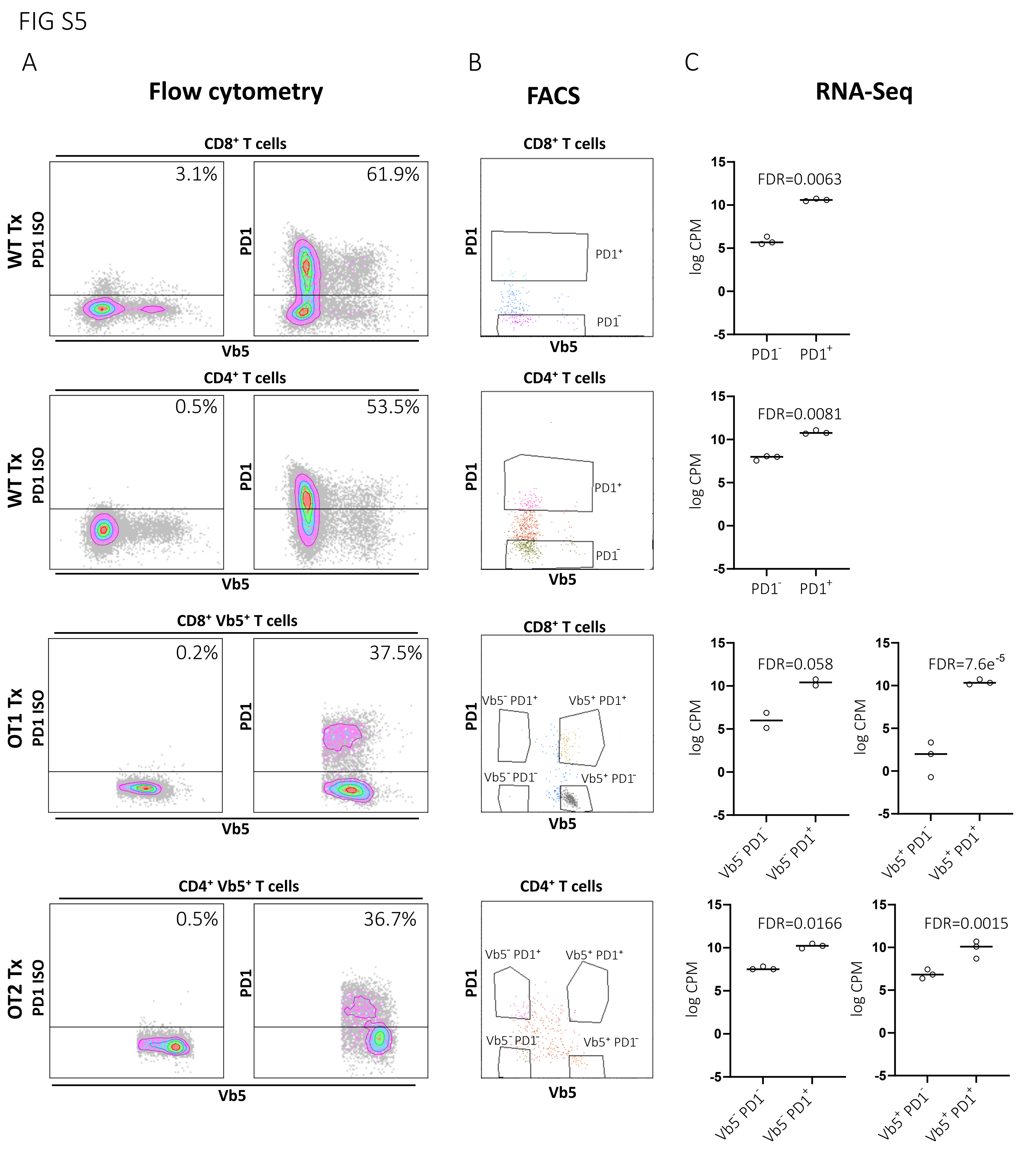
FIG S5
A
Flow cytometry
B
FACS
C
RNA-Seq
CD8⁺ T cells
3.1%
61.9%
WT Tx
PD1 ISO
PD1
Vb5
PD1⁺
PD1⁻
FDR=0.0063
log CPM
CD4⁺ T cells
0.5%
53.5%
FDR=0.0081
CD8⁺ Vb5⁺ T cells
0.2%
37.5%
OT1 Tx
Vb5⁻ PD1⁺
Vb5⁺ PD1⁺
Vb5⁻ PD1⁻
Vb5⁺ PD1⁻
FDR=0.058
FDR=7.6e-5
CD4⁺ Vb5⁺ T cells
0.5%
36.7%
OT2 Tx
FDR=0.0166
FDR=0.0015
