# Supplementary material for: Evidence for Non-Cancer-Specific T Cell Exhaustion in the Tcl1 Mouse Model for Chronic Lymphocytic Leukemia
Source: Int J Mol Sci. 2021 Jun 22;22(13):6648. doi: 10.3390/ijms22136648 (PMC8268419; doi:10.3390/ijms22136648)
Supplement: Supplementary file 1 [file ijms-22-06648-s001.zip › Supplementary Information.pdf]

Supplementary Information:

Supplemental Figure S1: T cell populations, survival and tumor development of CLL-transplanted OT1/OT2, OT1 Rag/OT2 Rag and WT mice.

Supplemental Figure S2: Depletion of T cells via pan B/CLL cell MACS isolation.

Supplemental Figure S3: Endogenous T cell expansion in peripheral blood and spleen after CLL transplantation.

Supplemental Figure S4: BCR analysis of CLL transplanted mice.

Supplemental Figure S5: PD1 expression based on flow cytometry, FACS and RNA-Seq analysis.

Supplemental Table S1: Differentially expressed genes (frd<0.05) from Volcano plots.
